# Comparison of SGLT2 inhibitors with DPP-4 inhibitors combined with metformin in patients with acute myocardial infarction and diabetes mellitus

**DOI:** 10.1186/s12933-023-01914-4

**Published:** 2023-07-22

**Authors:** Young Sang Lyu, Seok Oh, Jin Hwa Kim, Sang Yong Kim, Myung Ho Jeong

**Affiliations:** 1 Division of Endocrinology and Metabolism, Department of Internal Medicine, Chosun University Hospital, Gwangju, Republic of Korea; 2 Departmnent of Cardiology, Chonnam National University Hospital, Gwangju, Republic of Korea; 3 Department of Cardiology, Chonnam National University Medical School, Gwangju, Republic of Korea

**Keywords:** Antidiabetic agents, Diabetes mellitus, Hypoglycemic agents, Myocardial infarction

## Abstract

**Background:**

Although sodium-glucose cotransporter 2 inhibitors (SGLT2i) have demonstrated cardiovascular benefits in patients with type 2 diabetes mellitus, real-world evidence regarding their benefits to diabetic patients with acute myocardial infarction (AMI) is insufficient. This study evaluated cardiovascular outcomes by comparing SGLT2i with dipeptidyl peptidase-4 inhibitors (DPP-4i) in combination with metformin in diabetic patients with AMI.

**Methods:**

This study involved 779 diabetic participants with AMI from a Korean nationwide multicenter observational cohort, who were divided into two groups: (1) metformin plus SGLT2i group (SGLT2i group, *n* = 186) and (2) metformin plus DPP-4i (DPP-4i group, *n* = 593). The primary endpoint was one year of major adverse composite events (MACEs), a composite outcome of all-cause mortality, non-fatal myocardial infarction, any revascularization, cerebrovascular accident, and stent thrombosis. To balance the baseline differences, inverse probability of treatment weighting (IPTW) was performed.

**Results:**

After IPTW, the rate of MACEs in the SGLT2i group was not significantly lower than that in the DPP-4i group (hazard ratio [HR], 0.99; 95% confidence interval [Cl], 0.46 to 2.14, *p* = 0.983). In the unadjusted and adjusted analyses, all items for clinical outcomes were comparable between the two groups. In our exploratory analysis, the left ventricular ejection fraction showed a significant improvement in the SGLT2i group than in the DPP-4i group before achieving statistical balancing (6.10 ± 8.30 versus 2.95 ± 10.34, *p* = 0.007) and after IPTW adjustment (6.91 ± 8.91 versus 3.13 ± 10.41, *p* = 0.027).

**Conclusions:**

Our findings demonstrated that SGLT2i did not influence the rate of MACEs compared with DPP-4i in combination with metformin in diabetic patients with AMI but did improve left ventricular ejection fraction.

**Trial registration:**

Not applicable (retrospectively registered).

**Supplementary Information:**

The online version contains supplementary material available at 10.1186/s12933-023-01914-4.

## Background

Diabetes mellitus (DM) is the main risk factor for the onset of atherosclerotic cardiovascular disease (ASCVD). Conversely, cardiovascular disease is the leading cause of mortality in patients with DM [1]. Moreover, patients with DM are known to have poor short- and long-term prognoses following acute myocardial infarction (AMI) compared with those without DM [2]. Therefore, preventing myocardial infarction (MI) in diabetic patients is crucial, and if an AMI does occur, administering aggressive treatment is necessary to ensure optimal outcomes.

Combination therapy with oral hypoglycemic agents (OHAs) is frequently prescribed to optimize glucose control. Many clinicians use sodium-glucose cotransporter 2 inhibitors (SGLT2i) or dipeptidyl peptidase-4 inhibitors (DPP-4i) in patients with DM as a second-line therapy in combination with metformin (MET) monotherapy. However, these strategies have different mechanisms of action and clinical benefits. SGLT2i, a novel class of OHAs, reduce serum glucose levels via an insulin-independent mechanism by inhibiting the reabsorption of glucose from the proximal convoluted tubule in the kidney, which expedites renal glucosuria [3]. In recently published cardiovascular outcome trials (CVOTs), SGLT2i have proven cardiovascular benefits in patients with DM in both primary and secondary prevention [4–7], which is driven by a low incidence of cardiovascular death and hospitalization for heart failure (HF). Based on these results, the 2022 American Diabetes Association guidelines have recommended the use of SGLT2i in patients diagnosed with type 2 DM and who have a history of, or are at high risk for, ASCVDs and HF [8]. SGLT2i have been successful in improving glycemic control without causing hypoglycemia and effectively lowering body weight. DPP-4i have a different glucose-lowering effect, which works by deactivating the two main incretin hormones (i.e., glucose-dependent insulinotropic polypeptide and glucagon-like peptide-1). This leads to an increase in insulin and a decrease in glucagon [9]. DPP-4i have demonstrated neutral cardiovascular effects in CVOTs [10–13]. Nonetheless, in Korea, they are commonly used as second-line OHAs in combination with MET owing to their favorable tolerability, neutral effect on weight gain, and low risk of hypoglycemia [14–17].

In patients with AMI, the combination of OHAs appears to be an important strategy for achieving glycemic goals and long-term cardiovascular benefits. Determining the optimal combination of OHAs is important for the desired clinical outcomes of AMI. In a literature review, a few articles have compared the clinical outcomes of SGLT2i and DPP-4i [18–21]. However, most of these studies have focused on the investigation of surrogate markers of cardiovascular disease, such as metabolic risk factors, glycated hemoglobin (HbA1c), and lipid profiles, and have included the general population rather than specifically patients with AMI. Therefore, real-world evidence of a combination of OHAs in AMI patients is limited. This study aimed to evaluate cardiovascular outcomes by comparing the use of SGLT2i versus DPP-4i in combination with MET in patients with AMI and concomitant DM.

## Methods

### Data collection of study population and study scheme

All relevant information was gathered from the Korea Acute Myocardial Infarction Registry-V (KAMIR-V). The KAMIR-V is a nationwide, multicenter, observational cohort supported by the Korean Working Group of Acute Myocardial Infarction, which included Korean AMI patients from January 2016 to June 2020. In total, 43 tertiary medical facilities equipped with percutaneous coronary intervention (PCI) capabilities and on-site coronary artery bypass graft services participated in this registry [22, 23]. It gathers demographic and biological information including the characteristics and clinical outcomes of the Korean population with AMI. The study protocol was approved by the institutional review boards of the participating institutions.

**Fig. 1:**
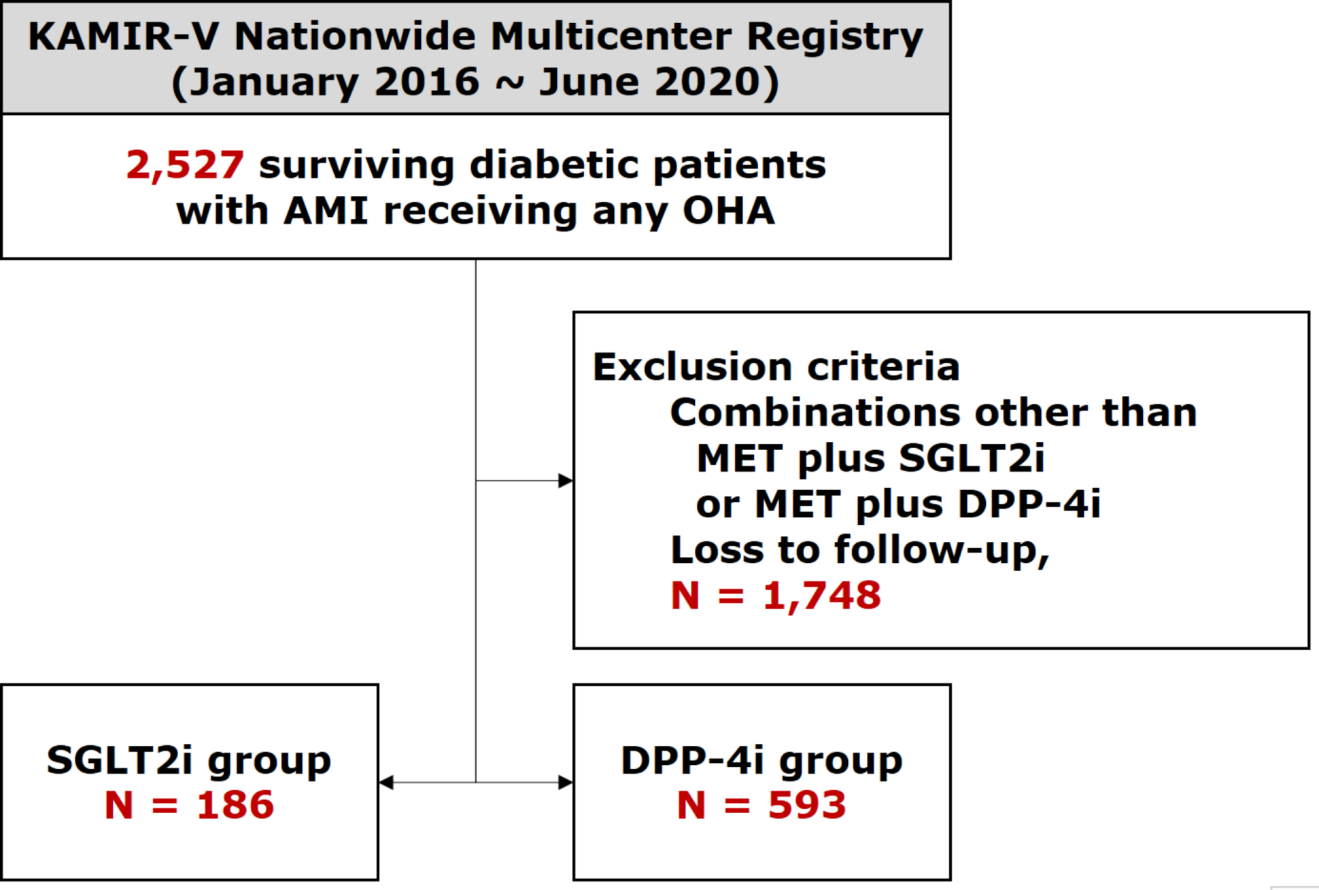
Study flowchart AMI = acute myocardial infarction; DPP-4i = dipeptidyl peptidase-4 inhibitors; KAMIR-V = Korea Acute Myocardial Infarction Registry-V; MET = metformin; OHA = oral hypoglycemic agent; SGLT2i = sodium-glucose cotransporter 2 inhibitors

Among a total of 15,628 cohort participants with suspected AMI in the KAMIR-V, 2,527 surviving participants with AMI and concomitant DM receiving any OHA were screened. After excluding (1) those who received combinations of OHA other than MET plus SGLT2i or MET plus DPP-4i and (2) those who were lost to follow-up, 779 participants were finally included in the analysis. These participants were subdivided into two groups based on their OHA types at the point of discharge: (1) MET plus SGLT2i group (SGLT2i group, *n* = 186) and (2) MET plus DPP-4i group (DPP-4i group, *n* = 593). The flowchart of the enrollment of participants is illustrated in Fig. 1.

### Definitions

According to the international guidelines [24], AMI is defined as myocardial injury characterized by a rise or fall in cardiac markers and related clinical manifestations as follows: (1) myocardial ischemia-related symptoms and/or signs; (2) new-onset changes in the 12-lead electrocardiogram (ECG), including deviated ST-segments (either elevation or depression), inverted T-waves, or the development of pathological Q-waves; (3) imaging evidence for loss of viable myocardium or regional wall motion abnormalities; or (4) existence of intracoronary thrombi during coronary angiography. ST-segment elevation MI (STEMI) is defined as AMI with new-onset ST-segment elevation in at least two continuous leads on the 12-lead surface ECG (> 0.2 mV in leads V1-3 or > 0.1 mV in all other leads on the 12-lead surface ECG) [25, 26]. As mentioned in previous studies [22, 27], the definitions of left main coronary artery (LMCA) disease and multivessel coronary artery disease (CAD) were defined as a disease with a ≥ 50% reduction in the diameter of LMCA and a disease with ≥ 70% reduction in the diameter of ≥ 2 native coronary vessels or ≥ 70% reduction in the diameter of one native coronary vessel with ≥ 50% reduction in the diameter of LMCA, respectively. Body mass index (BMI) was calculated using the mass (weight) and height values of the participants. Left ventricular ejection fraction (LVEF) was measured using 2-dimensional transthoracic echocardiography during hospitalization.

In addition, we recorded the angiographic and procedural characteristics in both groups. Intracoronary imaging guidance in PCI refers to the use of two currently available devices (intravascular ultrasound or optical coherence tomography) in the PCI procedure. The infarct-related artery, typically referred to as the culprit artery, is defined as a native coronary artery that is responsible for the development of AMI with occlusion or stenosis due to atherothrombotic changes. Thrombolysis In Myocardial Infarction (TIMI) flow grade was used as a quantitative and stratified indicator of antegrade intracoronary flow [28]. The characteristics of intracoronary lesions were defined according to the American College of Cardiology/American Heart Association classification (ACC/AHA) [29, 30]. Treatment strategies for PCI were subdivided as follows: (1) drug-eluting stent (DES) implantation, (2) bioresorbable vascular scaffold implantation, (3) balloon angioplasty only, and (4) other treatments. Infarct-related artery (IRA) was categorized into two types: (1) LMCA or left anterior descending coronary artery and (2) left circumflex coronary artery and right coronary artery.

### Clinical outcomes

Clinical follow-up of each participant was conducted for 12 months after enrollment. As previously described, it was performed at 6 months and 1 year through outpatient visits or whenever any cardiovascular event occurred [23]. The primary endpoint was a major adverse composite event (MACE), a composite outcome of all-cause mortality, non-fatal MI (NFMI), revascularization, cerebrovascular accident, and rehospitalization. The secondary endpoints included each individual component of MACE including all-cause mortality, cardiac/non-cardiac death, NFMI, any revascularization, cerebrovascular accident, and rehospitalization. Any revascularization refers to repeated PCI for any segment of the entire native coronary artery or coronary artery bypass graft. Rehospitalization was defined as the initial occurrence of an unplanned hospital admission due to different etiologies such as angina, HF, uncontrolled elevated blood glucose levels, hypoglycemia, or other factors.

### Exploratory outcomes

In addition to 1-year clinical outcomes, we performed a comparative analysis of the degree of improvement in left ventricular systolic function and glycemic control in patients following treatment in both groups. These investigations were based on the 1-year follow-up levels of LVEF and HbA1c and their changes from baseline during the 1-year follow-up period.

### Statistical analysis

All statistical analyses were conducted using SPSS (version 25.0; SPSS Inc., Armonk, New York, United States of America) and STATA (version 15.0; StataCorp, College Station, TX, USA). All continuous parameters were reported as means with standard deviations and examined by the Student’s t-test or Mann–Whitney test. All categorical (discrete) parameters were reported as frequencies with proportions (percentages) and examined by Pearson’s chi-square test or Fisher’s exact test.

The primary intention of this analysis was to determine whether there was a clear difference in clinical outcomes between the two combinations of OHAs (i.e., MET plus SGLT2i versus MET plus DPP-4i). To balance the baseline differences in this observational study, we employed a statistical matching technique, called inverse probability of treatment weighting (IPTW). In IPTW, the propensity score was constructed using multiple logistic regression analysis with the following baseline covariates: age, sex, use of emergency medical services, BMI, Killip classification, medical history, smoking history, family history of CAD, use of thrombolysis, LMCA disease, multivessel CAD, PCI, final diagnosis (STEMI or non-STEMI), initial HbA1c level, post-discharge medications, vascular approach (femoral or non-femoral), use of glycoprotein IIb/IIIa inhibitors, thrombus aspiration, intracoronary imaging guidance, preprocedural TIMI flow grade, ACC/AHA lesion characteristics, treatment strategies, and IRA.

We applied the Cox proportional hazard models to estimate hazard ratios and their corresponding 95% confidence intervals for each component of clinical outcomes, comparing DPP-4i group with SGLT2i group. In Cox models, we adjusted for all baseline covariates mentioned earlier.

In the present study, we plotted survival curves for MACEs using the Kaplan–Meier method. We also used the log-rank test to compare outcomes between the two groups. Participants with missing data for these baseline covariates or those who were lost to follow-up were precluded from these analyses.

## Results

### Baseline demographic and clinical characteristics

In this study, 779 participants with AMI and concomitant DM were included in the statistical analysis. In total, 186 (23.9%) and 593 (76.1%) subjects were allocated to SGLT2i group and DPP-4i group, respectively (Fig. 1).

All the relevant baseline characteristics are outlined in Tables 1 and 2. Patients in the SGLT2i group were more likely to be male, younger, and smokers than those in the DPP-4i group. Prior CAD was more frequent in the DPP-4i group than in the SGLT2i group. The SGLT2i group had more instances of STEMI as a final diagnosis than the DPP-4i group. Initial HbA1c levels were higher in the SGLT2i group than in the DPP-4i group. Among discharge medications, beta-blockers were more frequently prescribed to individuals in the SGLT2i group compared to those in the DPP-4i group. A comparison of the procedural characteristics showed that the SGLT2i group received more PCI with more DES implantation and a lower preprocedural TIMI flow grade than the DPP-4i group. All these differences were statistically balanced after the adjustment of baseline covariates with IPTW (Supplemental Table 1).

**Table 1:** Baseline clinical characteristics of the patients

	Before IPTW			After IPTW		
Characteristics	**SGLT2i group**	**DPP-4i group**	***p*** **-value**	**SGLT2i group**	**DPP-4i group**	***p*** **-value**
	**(n = 186)**	**(n = 593)**		**(n = 537)**	**(n = 532)**	
Male patients	150 (80.7)	422 (71.2)	**0.011**	358 (66.7)	388 (72.9)	0.364
Age, years	59.11 ± 11.52	66.12 ± 10.86	**< 0.001**	63.21 ± 11.17	63.74 ± 11.33	0.694
Age ≥ 75 years	20 (10.7)	153 (25.8)	**< 0.001**	76 (14.2)	105 (19.8)	0.269
EMS utilization	45 (24.2)	122 (20.6)	0.294	109 (20.2)	105 (19.7)	0.914
BMI ≥ 25 kg/m^2^	76 (43.4)	221 (40.0)	0.426	212 (39.4)	215 (40.4)	0.880
Killip class III-IV	17 (9.1)	51 (8.6)	0.830	52 (9.7)	55 (10.3)	0.877
Previous history						
Hypertension	113 (60.8)	395 (66.7)	0.136	350 (65.1)	344 (64.6)	0.935
DM	186 (100.0)	593 (100.0)	NA	537 (100.0)	532 (100.0)	NA
Dyslipidemia	48 (25.8)	134 (22.6)	0.367	125 (23.3)	133 (25.0)	0.741
Prior CAD	19 (10.2)	116 (19.6)	**0.003**	46 (8.6)	72 (13.5)	0.176
Prior heart failure	6 (3.2)	9 (1.5)	0.140	10 (1.8)	9 (1.8)	0.970
Prior CVA	12 (6.5)	56 (9.5)	0.214	33 (6.1)	49 (9.2)	0.310
DM duration			0.078			
0 to 10 years	98 (73.7)	259 (65.4)		NA	NA	NA
> 10 years	35 (26.3)	137 (34.6)		NA	NA	NA
Smoking	110 (61.1)	278 (48.7)	**0.004**	254 (47.2)	283 (53.2)	0.350
Family history of CAD	16 (8.9)	44 (7.6)	0.580	40 (7.5)	42 (7.9)	0.885
Use of thrombolysis	2 (1.1)	2 (0.3)	0.243	3 (0.5)	2 (0.4)	0.811
LVEF, %	51.07 ± 12.20	52.58 ± 11.40	0.126	53.04 ± 12.55	52.00 ± 11.57	0.502
LVEF < 40%	29 (15.8)	70 (12.2)	0.205	79 (14.7)	78 (14.7)	0.984
STEMI diagnosis	100 (53.8)	227 (38.3)	**< 0.001**	244 (45.5)	241 (45.3)	0.976
HbA1c, %	8.33 ± 1.87	7.70 ± 1.53	**< 0.001**	7.88 ± 1.65	7.85 ± 1.62	0.886
Discharge medications						
Aspirin	185 (99.5)	593 (100.0)	0.239	156 (99.7)	532 (100.0)	0.327
P2Y12 inhibitor	186 (100.0)	586 (98.8)	0.207	537 (100.0)	532 (100.0)	NA
Beta-blocker	163 (87.6)	455 (76.7)	**0.001**	431 (80.2)	416 (78.3)	0.734
ACE inhibitor or ARB	142 (76.3)	461 (77.7)	0.691	444 (82.5)	427 (80.3)	0.603
Statin	182 (97.9)	564 (95.1)	0.143	520 (96.7)	516 (97.0)	0.901
Ezetimibe	23 (12.4)	67 (11.3)	0.691	56 (10.5)	62 (11.6)	0.786

Values are presented as number (percentage) for categorical values and means ± standard deviation for continuous variables

ACE = angiotensin-converting enzyme; ARB = angiotensin receptor blocker; BMI = body-mass index; CAD = coronary artery disease; CVA = cerebrovascular accidents; DM = diabetes mellitus; DPP-4i = dipeptidyl peptidase-4 inhibitors; EMS = emergency medical service; HbA1c = hemoglobin A1c; IPTW = inverse probability of treatment weighting; LMCA = left main coronary artery; LVEF = left ventricular ejection fraction; NA = not applicable; PCI = percutaneous coronary intervention; SGLT2i = sodium-glucose cotransporter 2 inhibitors; STEMI = ST-segment elevation myocardial infarction

**Table 2:** Baseline procedural characteristics of the patients

	Before IPTW			After IPTW		
Characteristics	**SGLT2i group**	**DPP-4i group**	***p*** **-value**	**SGLT2i group**	**DPP-4i group**	***p*** **-value**
	**(n = 186)**	**(n = 593)**		**(n = 537)**	**(n = 532)**	
Use of PCI	184 (98.9)	551 (92.9)	**0.001**	537 (100.0)	532 (100.0)	NA
LMCA disease	11 (5.9)	38 (6.4)	0.797	33 (6.1)	33 (6.2)	0.978
Multivessel CAD	111 (59.7)	353 (59.8)	0.970	344 (64.1)	320 (60.1)	0.521
Use of transfemoral approach	88 (47.8)	248 (45.0)	0.507	241 (44.9)	246 (46.2)	0.851
Use of GPIIb/IIIa inhibitor	11 (6.0)	46 (8.3)	0.298	41 (7.7)	43 (8.0)	0.926
Use of thrombus aspiration	12 (6.5)	63 (11.4)	0.057	79 (14.7)	54 (10.2)	0.460
Intracoronary imaging guidance (OCT or IVUS)	63 (34.2)	154 (27.9)	0.105	151 (28.0)	163 (30.7)	0.627
Preprocedural TIMI flow grade 0-I	81 (44.0)	206 (35.6)	**0.041**	175 (32.5)	209 (39.2)	0.240
ACC/AHA lesion characteristics B2/C	151 (84.4)	444 (84.4)	0.987	471 (87.6)	448 (84.2)	0.344
Treatment strategies			**0.039**			0.160
DES implantation	178 (97.3)	523 (94.9)		528 (98.2)	512 (96.1)	
BVS implantation	1 (0.5)	1 (0.2)		1 (0.2)	0 (0.0)	
Balloon angioplasty only	3 (1.6)	27 (4.9)		7 (1.3)	20 (3.9)	
Others	1 (0.5)	0 (0.0)		1 (0.3)	0 (0.0)	
Infarct-related artery			0.306			0.614
LMCA or LAD	98 (53.6)	271 (49.2)		253 (47.1)	268 (50.4)	
LCX or RCA	85 (46.4)	280 (50.8)		284 (52.9)	264 (49.6)	

Values are presented as number (percentage) for categorical values and means ± standard deviation for continuous variables

Categorical values are presented as number (percentage)

ACC = American College of Cardiology; AHA = American Heart Association; BVS = bioresorbable vascular scaffold; DES = drug-eluting stent; DPP-4i = dipeptidyl peptidase-4 inhibitors; GPIIb/IIIa = glycoprotein IIb/IIIa; IVUS = intravascular ultrasound; LAD = left anterior descending coronary artery; LCX = left circumflex coronary artery; LMCA = left main coronary artery; NA = not applicable; OCT = optical coherence tomography; PCI = percutaneous coronary intervention; RCA = right coronary artery; SGLT2i = sodium-glucose cotransporter 2 inhibitors; TIMI = Thrombolysis in Myocardial Infarction

### Clinical and exploratory outcomes

**Fig. 2:**
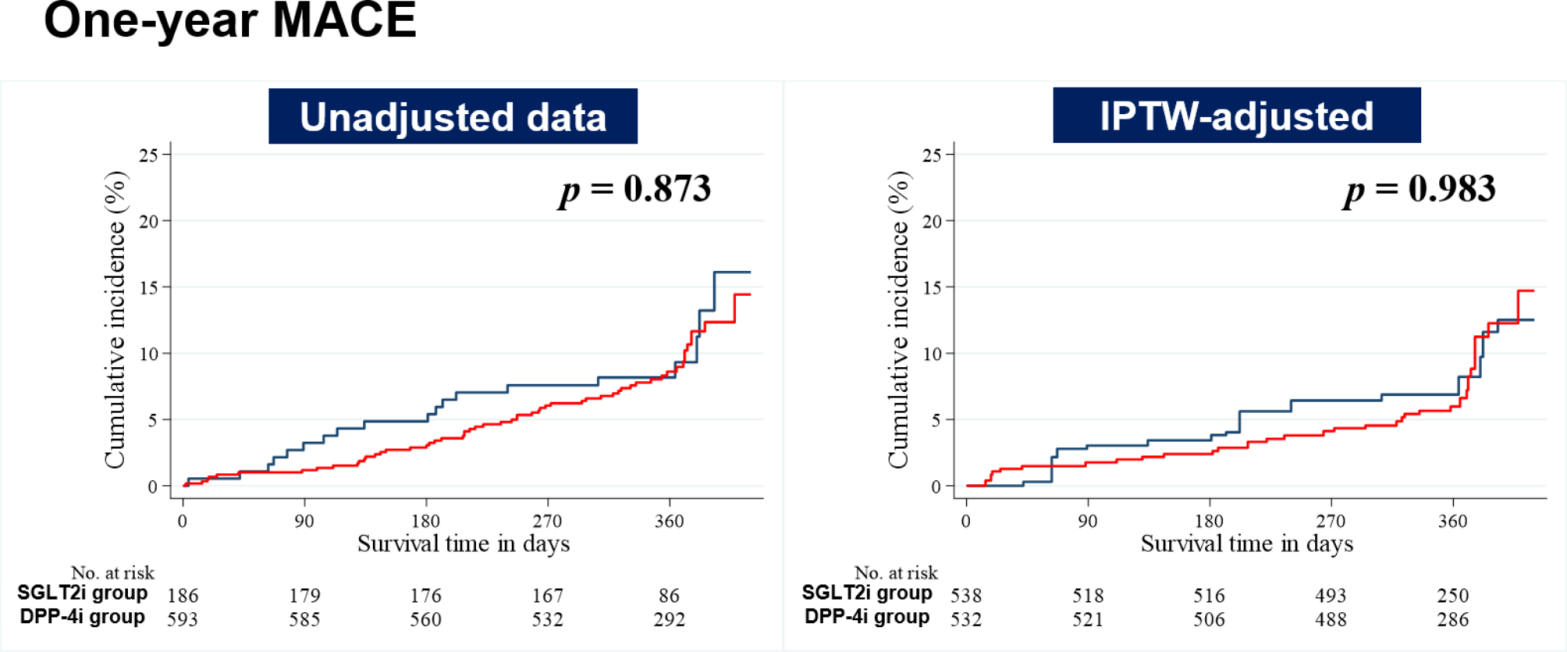
1-year event rates of MACE in the study population DPP-4i = dipeptidyl peptidase-4 inhibitors; IPTW = inverse probability of treatment weighting; MACE = major adverse composite event; SGLT2i = sodium-glucose cotransporter 2 inhibitors

The 1-year clinical outcomes of all participants are outlined in Table 3. The median follow-up interval was 361 days. These outcomes included MACE and its individual components including all-cause mortality (cardiac death and non-cardiac death), NFMI, revascularization, and rehospitalization. Kaplan–Meier survival curves of 1-year MACE were also illustrated in the unadjusted and IPTW-adjusted datasets (Fig. 2). In the unadjusted and IPTW-adjusted analyses, all items for clinical outcomes were comparable between the two groups.

We further investigated the exploratory outcomes according to LVEF and HbA1c levels, which are summarized in Table 4. Both HbA1c and LVEF levels at the 1-year follow-up were comparable between the groups. However, group A had lower Δ HbA1c but higher Δ LVEF than group B. These differences were statistically attenuated in the covariate-adjusted data, except for Δ LVEF after IPTW adjustment.

**Table 3:** 1-year clinical outcomes in propensity score matched patients

Outcomes	SGLT2i group	DPP-4i group	Unadjusted analysis		IPTW-adjusted analysis	
	**(n = 186)**	**(n = 593)**	**HR (95% CI)**	***p*** **-value**	**HR (95% CI)**	***p*** **-value**
MACE	19 (10.2)	58 (9.8)	0.96 (0.57–1.61)	0.873	0.99 (0.46–2.14)	0.983
All-cause mortality	0 (0.0)	11 (1.8)	NA	NA	NA	NA
Cardiac death	0 (0.0)	5 (0.8)	NA	NA	NA	NA
Non-cardiac death	0 (0.0)	6 (1.0)	NA	NA	NA	NA
Myocardial infarction	2 (1.1)	9 (1.5)	1.47 (0.32–6.82)	0.621	2.78 (0.31–24.90)	0.362
Any revascularization	12 (6.4)	22 (3.7)	0.56 (0.28–1.13)	0.108	0.68 (0.26–1.74)	0.418
CVA	4 (2.1)	8 (1.3)	0.67 (0.20–2.23)	0.513	1.43 (0.24–8.71)	0.697
Rehospitalization	3 (1.6)	19 (3.2)	2.05 (0.61–6.94)	0.247	1.58 (0.28–8.99)	0.603

Categorical values are presented as percentage (number)

CI = confidence interval; CVA = cerebrovascular accident; DPP-4i = dipeptidyl peptidase-4 inhibitors; HR = hazard ratio; NA = not applicable; IPTW = inverse probability of treatment weighting; MACE = major adverse composite event; SGLT2i = sodium-glucose cotransporter 2 inhibitors

**Table 4:** 1-year exploratory outcomes in propensity score matched patients

	Before IPTW			After IPTW		
Characteristics	**SGLT2i group**	**DPP-4i group**	***p*** **-value**	**SGLT2i group**	**DPP-4i group**	***p*** **-value**
	**(n = 186)**	**(n = 593)**		**(n = 537)**	**(n = 532)**	
1-year follow-up						
HbA1c, %	7.03 ± 1.39	7.08 ± 1.45	0.804	7.06 ± 1.18	6.95 ± 1.36	0.483
LVEF, %	53.97 ± 10.71	55.41 ± 10.12	0.284	56.41 ± 11.06	55.08 ± 10.29	0.448
LVEF < 40%	7 (9.2)	14 (5.4)	0.232	15 (8.0)	16 (5.9)	0.559
Change from baseline during 1-year follow-up						
Δ HbA1c	-1.15 ± 2.00	-0.65 ± 1.90	**0.041**	-0.76 ± 1.73	-0.80 ± 2.00	0.910
Δ LVEF	6.10 ± 8.30	2.95 ± 10.34	**0.007**	6.91 ± 8.91	3.13 ± 10.41	**0.027**

Values are presented as number (percentage) for categorical values and means ± standard deviation for continuous variables

DPP-4i = dipeptidyl peptidase-4 inhibitors; HbA1c = hemoglobin A1c; IPTW = inverse probability of treatment weighting; LVEF = left ventricular ejection fraction; SGLT2i = sodium-glucose cotransporter 2 inhibitors

## Discussion

This study aimed to compare the cardiovascular outcomes of SGLT2i and DPP-4i as add-on therapies in patients with AMI and concomitant DM receiving MET. Our results showed that SGLT2i use was not associated with a reduced risk of MACEs compared with DPP-4i use in patients with AMI. However, our study demonstrated the potential benefit of SGLT2i over DPP-4i in improving LVEF, implying that the use of SGLT2i may exert beneficial effects in the clinical setting of AMI, although it did not reduce MACE.

Considering the cardiovascular benefits of SGLT2i in diabetic patients as established in recently published CVOTs [4–7], guidelines recommend prioritizing the use of SGLT2i in patients with type 2 DM and concurrent cardiovascular disease. However, these CVOTs of SGLT2i enrolled subjects with chronic ASCVDs and not AMI; therefore, evidence of SGLT2i use in AMI and type 2 DM is still limited. Only one retrospective observational study evaluated patients with AMI and concomitant type 2 DM, revealing that the use of SGLT2i was related with a reduced risk of adverse cardiovascular events [31]. One limitation of this study was that the control group consisted of patients not taking SGLT2i rather than those taking an active competitor. Moreover, it was conducted at a single center in Taiwan and had a small sample size, which means that it is somewhat difficult to generalize their results. However, our study expanded on previous retrospective observational studies using active competitors such as DPP-4i prevalently described for use in type 2 DM patients and providing evidence in Korean patients using the KAMIR-V, a nationwide multicenter observational cohort of patients with AMI undergoing PCI.

Our study showed no significant difference in the incidence of cardiovascular events between SGLT2i and DPP-4i. However, the results of our study were significantly different from those of previous observational [32, 33] and network meta-analyses comparing SGLT2i and DPP-4i [34], demonstrating that SGLT2i have a beneficial effect on cardiovascular disease compared with DPP-4i. This disparity seems to be interesting and may be expounded by the different features of enrolled participants, such as the acute stage of MI in our study versus the chronic stage of MI in previous studies. In addition, it might not be sufficient to demonstrate statistical significance in our study because of the insufficient number of subjects and the relatively short follow-up period. Meanwhile, DPP-4i may have some beneficial effects on AMI, the acute stage of MI. This hypothesis has been supported in several studies. Wang et al. reported that DPP-4i therapy improved the three-year survival rates of patients with first-time AMI and concomitant DM [35]. Vildagliptin, a DPP-4i, reduced acute mortality in post-AMI and concomitant type 2 DM in a rat study by restoring the autophagic response through the attenuation of Bcl-2-Beclin-1 interaction [36]. However, explaining this clearly even with this assumption is difficult; therefore, further study is needed with a large number of subjects and long-term follow-up.

HF, a chronic complication of DM, is important for the prognosis of DM but has been underdiagnosed [37, 38]. The benefits of SGLT2i were first established in patients with HF with reduced ejection fraction [39, 40], and a recently published large-scale study revealed that these effects were also significant in patients with HF with preserved ejection fraction [41, 42]. Based on these studies, recent guidelines specify that SGLT2i should be considered as a first-line treatment for HF, regardless of ejection fraction [43, 44]. Similarly, in our study, LVEF was significantly improved in patients treated with SGLT2i than in patients treated with DPP-4i before achieving statistical balancing and after IPTW adjustment. This implies that SGLT2i use may achieve more improvement of left ventricular systolic function. These findings appear to be consistent with the beneficial effects of SGLT2 inhibitors on HF. This improvement in systolic function is indeed beneficial, particularly in patients with AMI who are at a high risk of HF.

Although not fully understood, the effect of SGLT2i on HF can be explained by several mechanisms in addition to hypoglycemic and diuretic effects. These theories include the following potential benefits of sodium-glucose cotransporter 2 inhibition: inhibiting the cardiac Na+/H + exchanger, decreasing oxidative stress, and improving cardiac energy metabolism. SGLT2i have been found to have an inhibitory effect on the Na+/H + exchanger in cardiac cells. The Na+/H + exchanger is a protein that regulates the exchange of sodium and hydrogen ions across cell membranes, including those of cardiac cells [45]. This exchange is essential for maintaining the proper intracellular pH and ion balance in the heart. In HF, the Na+/H + exchanger is frequently upregulated or overactivated leading to increased sodium and calcium levels within cardiac cells [46]. This elevated intracellular sodium and calcium can contribute to cellular dysfunction, impaired relaxation, and increased workload on the heart, further exacerbating HF. SGLT2i helps to restore the balance of sodium and calcium within cardiac cells by inhibiting the Na+/H + exchanger. This inhibition leads to a reduction in intracellular sodium and calcium levels, thereby improving cellular function and alleviating the workload on the heart [45, 47]. By targeting this specific mechanism, SGLT2i have the potential to ameliorate the adverse effects of Na+/H + exchanger overactivation in HF.

Chronic inflammation and increased oxidative stress also play a significant role in the development and progression of HF [48, 49]. SGLT2i have been shown to possess anti-inflammatory and antioxidant properties. These drugs can reduce the production of pro-inflammatory molecules [50, 51] and inhibit oxidative stress [52]. By dampening inflammation and oxidative stress, SGLT2i protect cardiac cells and mitigate the detrimental effects of HF [50, 52, 53]. Finally, HF is associated with alterations in cardiac energy metabolism. SGLT2i have been found to modulate cardiac metabolism by increasing reliance on fatty acids as an energy source and promoting ketone body utilization [54, 55]. This metabolic shift can improve energy efficiency, preserve myocardial function, and enhance overall cardiac performance in individuals with HF [56, 57].

Besides, despite SGLT2i, with their aforesaid cardiovascular benefits, are superior in the improvement in LVEF compared with DPP-4i, clinical outcomes became comparable in both groups before and after IPTW. As previously mentioned, this discrepancy may be explained by the relatively small sample size with the relatively short follow-up interval. Moreover, LVEF at baseline seemed to be relatively good with the inclusion of less than 20% of patients with LVEF < 40% in each group, which may significantly offset the superior cardiovascular safety of SGLT2i. Since this issue is still not fully explainable, further investigations should be performed in the future.

Our results demonstrated that DPP4-i had similar but numerically low risk of any revascularization to SGLT2i. It is still contentious whether DPP4-i have beneficial effects in terms of coronary revascularization or not. A meta-analysis of CVOTs for DPP-4i showed that DPP-4i resulted in a non-significant decrease in coronary revascularization [58], whereas some CVOTs on SGLT2i showed robust evidence for the prevention of HF but not for atherosclerotic changes such as coronary revascularization or peripheral revascularization [6, 59]. In contrast to these publications, recent evidence from administrative data in Taiwan revealed that the use of SGLT2i was associated with a lower risk of revascularization compared with the use of DPP-4i [60]. These disparities between the studies may be owing to the variations in study designs and patient enrollment criteria. Therefore, further investigations are needed to confirm the effect of SGLT2i versus DPP-4i on atherosclerotic changes, including coronary revascularization.

We should also note that less than a quarter of the study population received SGLT2i. Given that SGLT2i are one of promising classes of OHAs with well-established cardiovascular benefits [61, 62], their prescription was still insufficient compared with other cardio-protective medications. In the real-world practice, SGLT2i have been more under-prescribed than DPP-4i worldwide [16, 63]. In a Korean population-based cohort study, the prescription of SGLT2i has been suboptimal among patients with established ASCVDs or HF. Similarly, in data claims by the United States of America, patients were less likely to initiate treatment with SGLT2i than with DPP-4i [64]. These trends may be explained by the “occupation effect,” considering that DPP4-i were introduced in 2006 but SGLT2i in 2012 [63]. According to the Diabetes Fact Sheets in Korea, DPP-4i, one of previously established OHAs, still occupy a large proportion of total OHA market shares [17]. They are the third most common OHA monotherapy after MET and sulfonylurea, and the combination of both MET and DPP-4i has been the most common dual combination therapy since 2014. Furthermore, the use of DPP-4i may be one of independent factors affecting the non-initiation of SGLT2i [16]. This can be partly explained given the context that the combination of both SGLT2i and DPP-4i was not covered by the Korean national health insurance [16], as well as the “occupation effect” of DPP-4i over SGLT2i. Hence, the revision of healthcare insurance policy will be required to enable extensive insurance coverage of a combination of OHAs to bridge this evidence-practice gap in real-world practice.

Another notable finding is that the incidence of all-cause mortality was generally low in the present study. There are several possible explanations for this finding. First, as summarized in Tables 1 and 2, all participants received high rates of cardio-protective medications and high rates of PCI, which may account for their low mortality rate to some extent. Second, potential selection bias may influence these findings. As illustrated in the study scheme (Fig. 1), “survivor-cohort effect” may partially account for these findings, given that we included participants who survived during the index hospitalization. Furthermore, our study excluded patients who were lost to follow-up, which also contributed to the selection bias. That is, there may have been more deaths than were included.

To the best of our knowledge, there have been no head-to-head comparison studies of novel OHAs in the acute phase of MI. Therefore, our study offers important information in real-world settings and provides useful information when selecting a second-line OHA as add-on therapies with MET in patients who need additional glucose-lowering effects. An additional strength of our study is that we used the statistical matching technique, IPTW, to balance the different baseline characteristics.

### Study limitations

Our study had some key limitations. First, the KAMIR-V was conducted only in tertiary medical centers that treat high-volume AMI patients. Therefore, generalizing the clinical outcomes, including cardiovascular outcomes, to all medical institutions with patients experiencing AMI is difficult. Second, although this study was based only on a prospective, observational registry, it was a nonrandomized study. Despite employing two different propensity score weighting methods to reduce selection bias, the problem of selection bias may still persists owing to several reasons, such as data selection by inclusion and exclusion criteria, the presence of data with missing values, and the possibility of unmeasured confounders. Third, the patient follow-up period in our study was one year, which is a relatively short period for statistical significance. Fourth, several important factors that may affect clinical outcomes were unavailable, which could have been clinically crucial. That is, we could not collect detailed information about OHAs such as adherence to or duration of these medications, their initiation timing, their dosages, or transitions to another class of OHA. Furthermore, glucose-lowering medications used before enrollment are unknown; therefore, it is not possible to confirm the effect of starting a new diabetes drug. Fifth, as our study has a relatively low statistical power with a small sample size and it excludes the untreated group (patients treated with MET monotherapy) from the statistical analysis, large-scale randomized controlled trials should be conducted to perform a head-to-head comparison between drugs in the future.

## Conclusions

The use of SGLT2i compared with DPP-4i over one year did not demonstrate a statistically significant difference in composite cardiovascular outcomes in patients with AMI and concomitant DM. However, the use of SGLT2i appeared to improve left ventricular systolic function compared with the use of DPP-4i.

### Electronic supplementary material

Below is the link to the electronic supplementary material.

Supplementary Material 1

## Data Availability

The datasets used and/or analyzed during the current study are available from the corresponding author on reasonable request.

## Abbreviations

ACC/AHA: American College of Cardiology/American Heart Association classification
AMI: Acute myocardial infarction
ASCVD: Atherosclerotic cardiovascular disease
BMI: Body mass index
CAD: Coronary artery disease
CVOT: Cardiovascular outcome trials
DES: Drug-eluting stent
DM: Diabetes mellitus
DPP-4i: Dipeptidyl peptidase-4 inhibitors
ECG: Electrocardiogram
HbA1c: Glycated hemoglobin
HF: Heart failure
IPTW: Inverse probability of treatment weighting
KAMIR-V: Korea Acute Myocardial Infarction Registry-V
LMCA: Left main coronary artery
LVEF: Left ventricular ejection fraction
MACE: Major adverse composite event
MET: Metformin
MI: Myocardial infarction
NFMI: Non-fatal myocardial infarction
OHA: Oral hypoglycemic agent
PCI: Percutaneous coronary intervention
SGLT2i: Sodium-glucose cotransporter 2 inhibitors
STEMI: ST-segment elevation myocardial infarction
TIMI: Thrombolysis In Myocardial Infarction
