# Correction: Comparison of SGLT2 inhibitors with DPP4 inhibitors combined with metformin in patients with acute myocardial infarction and diabetes mellitus

**DOI:** 10.1186/s12933-023-01960-y

**Published:** 2023-09-05

**Authors:** Young Sang Lyu, Seok Oh, Jin Hwa Kim, Sang Yong Kim, Myung Ho Jeong

**Affiliations:** 1 Division of Endocrinology and Metabolism, Department of Internal Medicine, Chosun University Hospital, Gwangju, Republic of Korea; 2 Departmnent of Cardiology, Chonnam National University Hospital, Gwangju, Republic of Korea; 3 Department of Cardiology, Chonnam National University Medical School, Gwangju, Republic of Korea

**Correction to: Cardiovascular Diabetology (2023) 22:185**

10.1186/s12933-023-01914-4

Following publication of the original article [1], the author noticed an error in the textual part under Study Limitations section. This has now been corrected with this erratum.

The sentence should read, “Despite employing a different propensity score weighting method to reduce selection bias, the problem of selection bias may still persists owing to several reasons, such as data selection by inclusion and exclusion criteria, the presence of data with missing values, and the possibility of unmeasured confounders.“ instead of “Despite employing two different propensity score weighting methods to reduce selection bias, the problem of selection bias may still persists owing to several reasons, such as data selection by inclusion and exclusion criteria, the presence of data with missing values, and the possibility of unmeasured confounders.“
