# Supplementary material for: Comparison of SGLT2 inhibitors with DPP-4 inhibitors combined with metformin in patients with acute myocardial infarction and diabetes mellitus
Source: Cardiovasc Diabetol. 2023 Jul 22;22:185. doi: 10.1186/s12933-023-01914-4 (PMC10362625; doi:10.1186/s12933-023-01914-4)
Supplement: Supplementary file 1 — Supplementary Material 1 [file 12933_2023_1914_MOESM1_ESM.docx]

**Supplemental Table 1.** Distribution of absolute standardized mean differences for each covariate used in the derivation of the propensity scores.

|  | Standardized mean differences | |
| --- | --- | --- |
|  | **Unadjusted data** | **IPTW-adjusted data** |
| Baseline clinical characteristics | | |
| Age ≥ 75 years | 0.6532 | 0.0457 |
| Male patients | 0.2089 | 0.1281 |
| EMS utilization | 0.1391 | 0.0191 |
| BMI ≥ 25 kg/m^2^ | 0.0294 | 0.0225 |
| Killip class III-IV | 0.0700 | 0.0079 |
| Previous history |  |  |
| Hypertension | 0.1756 | 0.0114 |
| Diabetes mellitus | NA | NA |
| Dyslipidemia | 0.0853 | 0.0385 |
| Prior CAD | 0.1471 | 0.1522 |
| Prior heart failure | 0.1663 | 0.0026 |
| Prior CVA | 0.2090 | 0.1217 |
| Smoking | 0.2377 | 0.1155 |
| Family history of CAD | 0.1064 | 0.0133 |
| Use of thrombolysis | 0.1293 | 0.0201 |
| LVEF, % | 0.1981 | 0.0780 |
| LVEF <40% | 0.1817 | 0.0081 |
| STEMI diagnosis | 0.2451 | 0.0067 |
| HbA1c, % | 0.4816 | 0.0233 |
| Discharge medications |  |  |
| Aspirin | NA | NA |
| P2Y12 inhibitor | NA | NA |
| Beta-blocker | 0.3118 | 0.0397 |
| ACE inhibitor or ARB | 0.1031 | 0.0502 |
| Statin | 0.0094 | 0.0077 |
| Ezetimibe | 0.0979 | 0.0365 |
| Baseline procedural characteristics | | |
| Use of PCI | NA | NA |
| LMCA disease | 0.0825 | 0.0060 |
| Multivessel CAD | 0.0731 | 0.0722 |
| Use of transfemoral approach | 0.1441 | 0.0268 |
| Use of GPIIb/IIIa inhibitor | 0.1104 | 0.0082 |
| Use of thrombus aspiration | 0.2479 | 0.1137 |
| Intracoronary imaging guidance | 0.0858 | 0.0447 |
| Infarct-related artery | 0.0874 | 0.0559 |
| ACC/AHA lesion characteristics B2/C | 0.0231 | 0.0934 |
| Preprocedural TIMI flow grade 0-I | 0.0911 | 0.1300 |
| Treatment strategies | 0.0990 | 0.1240 |

In the matched cohort, the covariates were more balanced between the two groups with all standardized differences below the 0.25 threshold.

ACC/AHA = American College of Cardiology/American Heart Association; ACE = angiotensin-converting enzyme; ARB = angiotensin receptor blocker; BMI = body mass index; CAD = coronary artery disease; CVA = cerebrovascular accidents; EMS = emergency medical service; GPIIb/IIIa = glycoprotein IIb/IIIa; HbA1c = hemoglobin A1c; IPTW = inverse probability of treatment weighting; LMCA = left main coronary artery; LVEF = left ventricular ejection fraction; NA = not applicable; PCI = percutaneous coronary intervention; STEMI = ST-segment elevation myocardial infarction; TIMI = Thrombolysis in Myocardial Infarction.
